# The Outcome of Porcine Foetal Infection with Bungowannah Virus Is Dependent on the Stage of Gestation at Which Infection Occurs. Part 1: Serology and Virology

**DOI:** 10.3390/v12060691

**Published:** 2020-06-26

**Authors:** Deborah S. Finlaison, Peter D. Kirkland

**Affiliations:** Virology Laboratory, Elizabeth Macarthur Agricultural Institute, New South Wales Department of Primary Industries, Menangle, NSW 2568, Australia; peter.kirkland@dpi.nsw.gov.au

**Keywords:** Bungowannah virus, foetus, pestivirus, porcine, real-time PCR, serology, virology

## Abstract

Bungowannah virus is a novel porcine pestivirus identified in a disease outbreak in Australia in 2003. The aim of this study was to determine the outcome of infection of the pregnant pig with this virus. Twenty-four pregnant pigs were infected at days 35, 55, 75 or 90 of gestation. Blood, tonsillar and rectal swabs were collected from each pig at birth and then weekly until euthanasia or death. Tissues were sampled at necropsy. Viral load was measured by real-time reverse-transcription polymerase chain reaction (qRT-PCR) and antibody levels in serum by peroxidase-linked immunoassay. Bungowannah virus was detected in the serum and excretions of all infected pigs at birth regardless of the stage of gestation at which infection occurred. Persistent infections occurred following infection prior to the development of foetal immunocompetence. Unexpectedly some animals infected at day 55 of gestation later cleared the virus and seroconverted. Viraemia and viral shedding resolved quickest following infection in late gestation.

## 1. Introduction

Bungowannah virus is a novel pestivirus identified from an outbreak of disease in a piggery in New South Wales, Australia, in June 2003 [1]. It is genetically distinct from the other recognised pestiviruses of pigs, classical swine fever virus (CSFV) and atypical porcine pestivirus (APPV) [2,3] with its closest genetic relationship to the recently identified Linda virus [4]. The disease was referred to as the porcine myocarditis syndrome, or PMC, because histological changes in affected animals consist almost exclusively of a multifocal non-suppurative myocarditis, with myonecrosis in some cases. The outbreak initially presented as sudden death in 2- to 3-week-old weaning age pigs, but soon after the onset there was a marked increase in the birth of stillborn foetuses and a slight increase in the occurrence of mummified pigs. Cumulative losses in some weeks exceeded 50% of pigs born, and it is estimated that as many as 50,000 pigs died in the initial outbreak. Due to the reproductive effects and disease occurring almost exclusively in the first 2–3 weeks of life it was presumed to be predominantly the consequence of in utero infection. This hypothesis was supported by the detection of elevated serum IgG levels in up to 50% of stillborn pigs and by the absence of disease in pigs soon after weaning or in sows farrowing affected litters [1].

The pestiviruses are well recognised reproductive pathogens where the outcome of infection is dependent on a number of factors including the pathogenicity of the infecting strain, the stage of gestation that infection occurs in relation to organogenesis and development of immune competence, where infection prior to foetal immunocompetence may result in a persistent infection due to immunotolerance [5,6,7,8,9,10,11,12,13,14]. Persistently infected (PI) animals remain serologically negative and demonstrate cell-mediated unresponsiveness to the infecting strain, shed virus throughout their lives, and are usually epidemiologically more important in ongoing virus transmission than acute, transiently infected animals [5,13,14,15,16,17,18,19,20,21,22]. It has been shown experimentally that pigs infected post-natally with Bungowannah virus develop transient infections that resolve over a 10-day period and transmit the virus inefficiently [23]. PI animals are usually the reservoirs of pestiviruses in nature but no PI pigs surviving past 6–8 weeks old have been identified in the affected piggery. Therefore, to better understand how virus transmission is maintained in an infected population it is important to know if long-term infections can occur and to clarify the significance and sources of viral shedding following in utero infections.

This study examined the virological and serological characteristics of in utero infection of the porcine foetus with Bungowannah virus at different stages of gestation. The primary objectives were to determine:

1. If the porcine foetus becomes infected in utero following intra-nasal exposure of the sow;
2. The concentrations of Bungowannah virus RNA in serum, and shed in oropharyngeal secretions and faeces, and whether this is affected by the stage of pregnancy at which the sow is infected;
3. If the pig foetus mounts a humoral immune response following in utero infection;
4. If persistent infections with Bungowannah virus occur and whether there is a critical stage of gestation at which infection results in this outcome;
5. The optimal tissue samples for the detection of Bungowannah virus;
6. If PI pigs can readily transmit infection to naïve pigs.

Infection of the porcine foetus with Bungowannah virus was successful, and the virological and serological characteristics following in utero infection at different stages of gestation are reported. The clinical signs and gross pathology are described in an accompanying manuscript [24].

## 2. Materials and Methods

### 2.1. Londitudinal Study Design

Twenty-four pregnant pigs (22 gilts and two parity-1 sows) with known joining dates were obtained from a piggery known to be free of Bungowannah virus, and sampled and shown to be seronegative on the day of inoculation. Pregnancy was confirmed by ultrasound examination prior to selection for the study. Gilts from this piggery were routinely vaccinated against parvovirus, leptospirosis, erysipelas and *E. coli* with commercial vaccines at selection and again 4 weeks later. Both gilts and sows were also vaccinated against leptospirosis, erysipelas and E. coli at 13 weeks of pregnancy. The animals were moved to facilities at the Elizabeth Macarthur Agricultural Institute 3–5 days before they were due to be infected.

The pigs were challenged intranasally at approximately day 35 (34–36), 55 (55–58), 75 (72–76) or 90 (90–92) of gestation (n = 6 per group referred to as D35, D55, D75 and D90) [24]. These time-points were selected as they were similar to those used in a previous study where foetuses were directly inoculated [25] and they span the gestational age at which the pig foetus is considered to become immunocompetent (70 days). Due to animal accommodation availability and the logistics of undertaking a study of this size, the four treatment groups were challenged and managed separately. The four batch farrowings occurred over a 5-month period. To facilitate intranasal challenge, the pregnant pigs were sedated approximately 30 minutes prior with Azaperone (40 mg/mL—up to 2 mL/20 kg).

All pregnancies were allowed to proceed to 113 days of gestation when all pigs were induced to farrow on day 114 to optimise collection of blood from pigs prior to suckling. Farrowing was induced with 500 µg cloprostenol given intramuscularly on the morning of day 113 and, if required, followed 24 h later with Oxytocin (10 i.u.) intramuscularly.

Pigs from infected litters were weaned when 21–25 days old. As the presence of Bungowannah virus could still be detected at weaning in several challenge groups, as many pigs as the secure containment facilities could hold were weaned and retained until at least 5 to 8 weeks of age (D35, n = 11; D55, n = 22; D75, n = 23; D90, n = 20). Selected pigs from D35 and D55 were kept in the study for a longer period of time to allow ongoing monitoring of virological and serological parameters and clinical signs [24]. Throughout the study, pigs that were moribund, not feeding or did not appear to be viable were euthanased by an intravenous overdose of pentobarbitone sodium.

Pigs from the three litters that did not become infected with Bungowannah virus (on the basis that the virus could not be detected in serum or body fluid for any of the pigs in the litter in a real-time reverse-transcription polymerase chain reaction (qRT-PCR) assay and negative precolostral serology results) were kept as control pigs until they were 14 to 21 days old, at which time they were euthanased.

Individual pig identifications (IDs) have been retained in the text where relevant to facilitate comparison between virological, serological, clinical signs, gross [24] and histopathology findings described in related manuscripts. The first number relates to the litter ID and the second the animal ID within the litter (generally in order of birth) e.g., 8-01 indicates the first piglet to be born in litter 8.

The animal studies were approved by the Animal Ethics Committee of the Elizabeth Macarthur Agricultural Institute, AEC Reference No. M09/02 (6 March 2009).

### 2.2. Inoculum

The pregnant pigs were challenged with approximately 5.3–5.8 log_10_ TCID_50_ of Bungowannah virus in 5 mL of phosphate buffered gelatin saline (PBGS; pH 7.3, 2.5 mL per nostril), with the exception of Litter 11 where the sow only received approximately 3.8 log_10_ TCID_50_ of virus. This inoculum was prepared as previously described [23] and confirmed to be free of porcine parvovirus, porcine circovirus type 2 and other pestiviruses by PCR [26,27]. Testing for porcine reproductive and respiratory syndrome virus was not performed because Australia is free of this virus.

### 2.3. Sample Collection—Pregnant Animals

A vaginal swab was collected from each sow on the day of farrowing. In addition, a piece of placenta was collected and swabbed. Vaginal swabs were then collected every 2–3 days until 14 days post-farrowing and then once to twice weekly until 21–28 days.

All swabs collected during the course of the study were placed in 2 mL of PBGS and stored at 4 °C prior to testing.

### 2.4. Sample Collection—Piglets

A clotted blood sample, and oropharyngeal and rectal swabs were collected from pigs shortly after birth and where possible before they suckled. While this was achieved in most instances, some animals farrowed earlier than 114 days or commenced farrowing earlier than 24 h after receiving the cloprostenol injection and had suckled. For pigs that were stillborn, body cavity fluid (in preferential order of pericardial>pleural>abdominal fluid) was collected rather than serum.

Thereafter, oropharyngeal and rectal swabs and clotted blood samples were collected weekly up to 8 weeks of age. After this time-point, any remaining pigs were sampled every 10–14 days up to 3 months of age. Three animals from the D55 group that were seronegative at birth were followed for an extended period (10-1 to 189 days, and 8-01 and 8-05 to 329 days of age). Urine was collected opportunistically from weaned pigs.

All pigs were subjected to a detailed necropsy and a wide range of samples were collected for virology, serology and histopathology. Samples for qRT-PCR were collected by firmly rubbing a swab across the freshly cut surface of a section of heart, lung, thymus, spleen, small intestine, inguinal lymph node and brain, and from the surface of the tonsil from pigs of infected litters; from pigs of uninfected litters, swabs were collected directly from the surface of the tonsil and from a cut section of lung and spleen.

### 2.5. Transmission Study Design

Two 5-and-a-half week-old pigs were obtained from the same piggery as the pregnant animals. After an acclimatisation period of 2 days in isolation, the two naïve pigs were housed for 28 days in the same room and pen with two pigs from D55 (8-01 and 10-01) considered PI with Bungowannah virus. The PI pigs were selected for the study based on low pre-suckle IgG levels (448 and 135 μg/mL), and high quantities of Bungowannah virus RNA in serum (6.9 and 5.5 log_10_ copies/mL serum) and on oropharyngeal swabs (6.1 and 6.8 log_10_ copies/swab) at 6 weeks of age compared with cohorts. Retrospective testing demonstrated both were seronegative for Bungowannah virus antibodies at birth, when tested in the peroxidase-linked immunoassay.

### 2.6. Sample Collection—Transmission Study

Serum, oropharyngeal and rectal swabs were collected from the introduced pigs on day 0, and then from the introduced and PI pigs on days 1, 8 (serum) or 9 (oropharyngeal and rectal swabs), 15, 22 and 28 days post-introduction.

### 2.7. RNA Extraction and Real-Time Reverse-Transcription Polymerase Chain Reaction (qRT-PCR)

Total nucleic acid was extracted from 50 uL of sera and PBGS containing swabs using the MagMax™-96 Viral RNA Isolation Kit (Ambion, Austin, Texas Cat. No. 1836). A Kingfisher® 96 magnetic particle handling system (Thermo Fisher Scientific, Waltham, MA, USA) was used for the extraction process and the purified nucleic acid was eluted in 50 µL of buffer.

qRT-PCR was used for the detection and quantification of Bungowannah virus RNA. Five microlitres of nucleic acid was amplified using the AgPath-ID™ One-Step RT-PCR kit (Applied Biosystems, Foster City, CA, USA, Cat No. 4387424) with primers and probe as previously published [28]. The reaction was run in an Applied Biosystems 7500 Fast or 7900HT Real-Time PCR System (Foster City, CA, USA) both run in standard mode. A 10-fold dilution series of Bungowannah virus RNA standards ranging from 10^7^–10^2^ RNA copies/5 µL [25] was included in each assay and the quantity of Bungowannah virus RNA in a sample determined from the standard curve. For quantification purposes 2.3 and 2.6 log_10_ copies/mL were considered the sensitivity of the assay for serum and swabs/tissues respectively although the accuracy of quantification was considered likely to be variable below 4.3 or 4.6 log_10_ copies/mL. Additionally, means were calculated against a baseline of 2.3 or 2.6 log_10_ copies/mL for negative samples depending on the sample type.

### 2.8. Serology

A peroxidase-linked immunoassay was used for the detection of Bungowannah virus antibodies [28]. Where an animal recorded an antibody titre ≥5120, a titre of 5120 (12.3 log_2_) was used for calculation of means; where no antibody was detected, a titre of 8 (3 log_2_) was assigned for calculation of means.

### 2.9. Analysis of Results

To facilitate analysis of data, Bungowannah qRT-PCR results for serum, oropharyngeal and rectal swabs for each challenge time-point were grouped into time intervals based on the age of the pigs (0–1, 2–9, 10–17, 18–24, 25–31, 32–38, 39–45, 46–52, 53–60, 61–70 and 71–80 days). The same process was applied for serology results although the first time intervals were 0 and 1–9 days. Pigs that where known to have suckled prior to Day 0 sampling were classified as having been sampled on day 1 for the purpose of this study. Where an animal had been sampled twice within one of these intervals, the mean result for that animal was used when calculating the time-point mean. In addition, the results for animals in the D35 and D55 challenge groups were subdivided based on the presence or absence of antibodies to Bungowannah virus in samples collected prior to suckling.

## 3. Results

### 3.1. Longitudinal Study

#### 3.1.1. Pregnant Pigs

**Figure 1:**
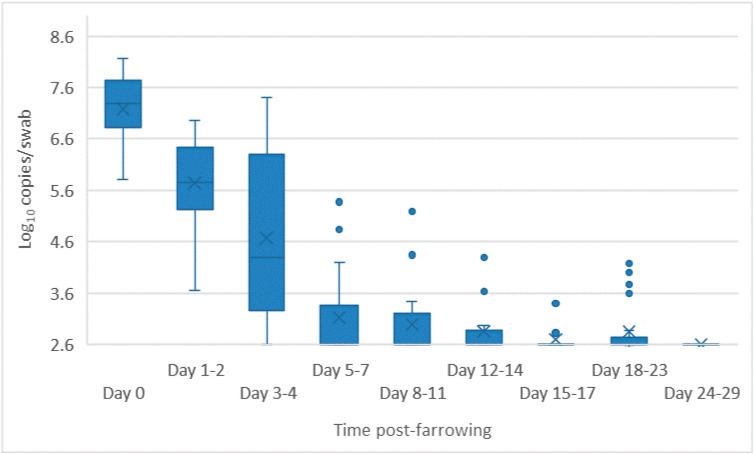
Box and whisker plot summarising the quantity of Bungowannah virus detected in vaginal swabs between 0–29 days post-farrowing following in utero infection (x = mean; — = median; the whiskers extend up from the top of the box to the largest data element that is less than or equal to 1.5 times the interquartile range (IQR) and down from the bottom of the box to the smallest data element that is larger than 1.5 times the IQR. Values outside this range are considered outliers and are represented by dots).

All pregnant pigs became infected with Bungowannah virus (as confirmed by seroconversion), with transplacental infection detected for 20 of the 23 (87%) litters. Transplacental infection did not occur for one pregnant pig in each of the D35, D55 and D75 challenge groups [24]. One pig from D55 was found to be not pregnant. For each sow that produced an infected litter, virus was detected in high quantities on vaginal swabs (mean 7.2 log_10_ copies/swab; range 5.8–8.2 log_10_ copies/swab) on the day of farrowing, decreasing rapidly by 5–7 days post-farrowing (mean 3.3 log_10_ copies/swab; range <2.6–5.4 log_10_ copies/swab) (Figure 1). By day 12–14 post-farrowing the mean amount of virus detected was 2.9 log_10_ copies/swab (range <2.6–4.3 log_10_ copies/swab). Viral RNA was not detected on any vaginal swabs collected after 23 days post-farrowing (n = 15). The mean quantity of Bungowannah virus RNA detected on placental swabs at day 0 (7.0 log_10_ copies/swab; range 5.7–8.1 log_10_ copies/swab) from the infected litters was comparable to that detected on vaginal swabs. Bungowannah virus RNA was not detected on vaginal or placental swabs collected from sows farrowing uninfected litters.

#### 3.1.2. Piglets—qRT-PCR

Bungowannah virus RNA was detected in the serum, body cavity fluid or internal tissues of 225/226 pigs in the 20 infected litters at birth regardless of the stage of gestation that the dam had been infected. It was concluded that one pig (11-15) had not become infected in utero based on the absence of viraemia at birth and because it became seronegative at 9 weeks old indicating the absence of a humoral immune response to infection. This pig was born after it was believed farrowing had concluded and had an antibody titre of ≥10,240 when sampled the next day, presumably due to ingestion of colostrum. No viraemia was detected during the 10 weeks it was followed in the study.

All pigs from three litters (n = 42) were uninfected at birth (based on the absence of viraemia and seronegative serology at birth). The pigs in these three litters all developed high antibody titres after ingestion of colostrum. They did not become infected with Bungowannah virus (based on the absence of a viraemia) despite being in the same room as pigs from infected litters where no direct contact was possible. Occasionally low quantities of Bungowannah virus were detected on oropharyngeal (n = 4; mean 3.5 log_10_ copies/swab; range 3.3–3.9 log_10_ copies/swab) and rectal swabs (n = 7; mean 3.2 log_10_ copies/swab; range 2.7–4.9 log_10_ copies/swab), presumably due to environmental contamination as a result of sharing a room with an infected litter. Viral RNA was never detected on oropharyngeal swabs from the animal in which 4.9 log_10_ copies were detected on the rectal swab, or on tissue samples at necropsy. Additionally, all tissue samples collected from these pigs were negative for Bungowannah virus RNA.

Results for the D35 and D55 groups have been subdivided based on whether Bungowannah virus antibodies were detected at birth (Ab +ve) or not (Ab −ve). Samples were collected from all weaned/surviving pigs in the D35, D75 and D90 groups until 75, 56 and 35 days of age respectively. In some cases on the day of euthanasia a direct tonsillar swab was collected rather than an oropharyngeal swab. This result is captured under the results for tissues. In the D55 group, samples were collected through until 120 days in the Ab +ve group (n = 4) and to 329 days in the Ab −ve group (n = 2; 8-01 and 8-05). The number of animals from which serum or body fluid was collected at each time-point for Bungowannah virus PCR is summarised in Table S1.

**Figure 2:**
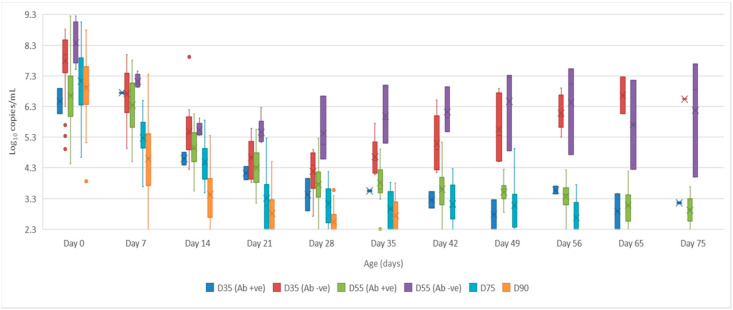
Box and whisker plot summarising the quantity of Bungowannah virus detected in serum between 0 to 75 days of age following in utero infection at one of four stages of gestation (x = mean; — = median; the whiskers extend up from the top of the box to the largest data element that is less than or equal to 1.5 times the interquartile range (IQR) and down from the bottom of the box to the smallest data element that is larger than 1.5 times the IQR. Values outside this range are considered outliers and are represented by dots).

**Figure 3:**
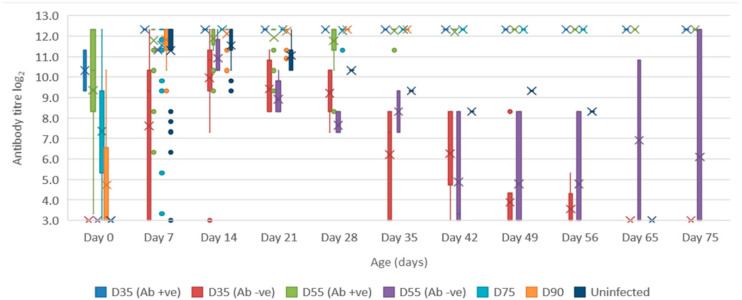
Box and whisker plot summarising the antibody titre against Bungowannah virus in serum as measured by peroxidase-linked immune assay between 0 to 75 days of age following in utero infection at one of four stages of gestation (x = mean; — = median; the whiskers extend up from the top of the box to the largest data element that is less than or equal to 1.5 times the interquartile range (IQR) and down from the bottom of the box to the smallest data element that is larger than 1.5 times the IQR. Values outside this range are considered outliers and are represented by dots).

**Figure 4:**
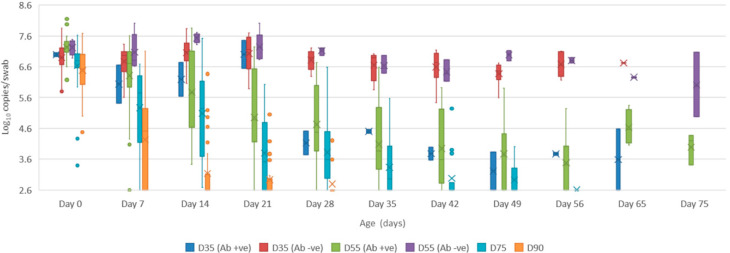
Box and whisker plot summarising the quantity of Bungowannah virus detected on oropharyngeal swabs between 0 to 75 days of age following in utero infection at one of four stages of gestation (x = mean; — = median; the whiskers extend up from the top of the box to the largest data element that is less than or equal to 1.5 times the interquartile range (IQR) and down from the bottom of the box to the smallest data element that is larger than 1.5 times the IQR. Values outside this range are considered outliers and are represented by dots).

**Figure 5:**
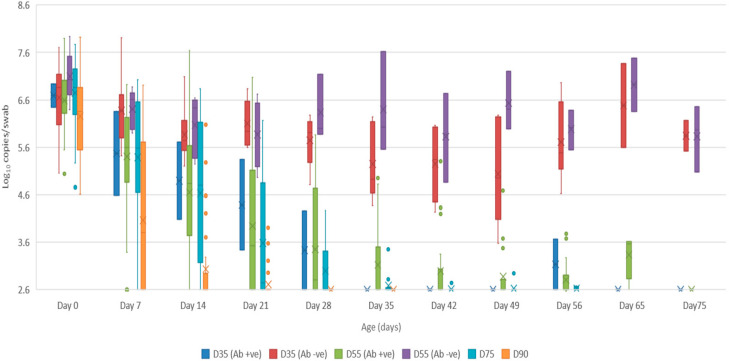
Box and whisker plot summarising the quantity of Bungowannah virus detected on rectal swabs between 0 to 75 days of age following in utero infection at one of four stages of gestation (x = mean; — = median; the whiskers extend up from the top of the box to the largest data element that is less than or equal to 1.5 times the interquartile range (IQR) and down from the bottom of the box to the smallest data element that is larger than 1.5 times the IQR. Values outside this range are considered outliers and are represented by dots).

**Figure 6:**
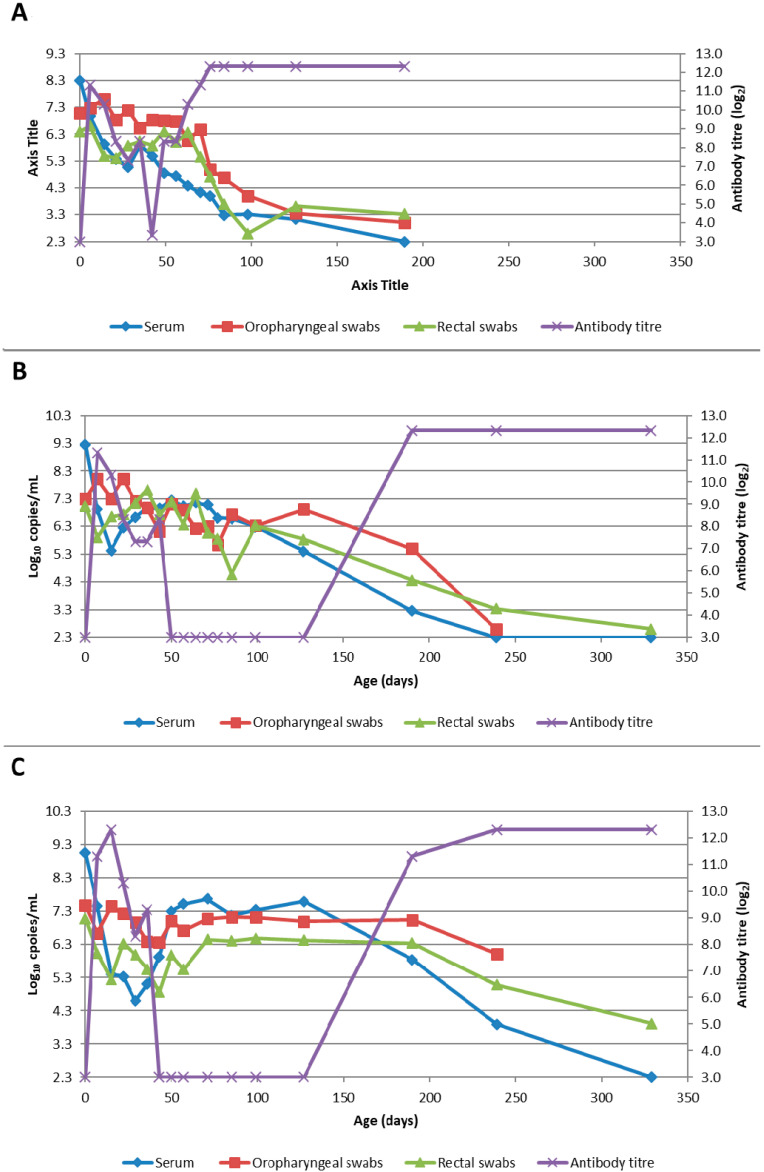
Quantity of Bungowannah virus detected in serum, oropharyngeal and rectal swabs, and antibody titre against Bungowannah virus detected in: (**A**) 10-01 from birth to 189 days of age; (**B**) 8-01 (D55 Ab −ve) from birth to 329 days of age; (**C**) 8-05 (D55 Ab −ve) from birth to 329 days of age.

- Serum: the mean quantity of Bungowannah virus RNA detected in serum at birth exceeded 6.4 log_10_ copies/mL for all groups of pigs, with the highest levels recorded in those animals where no antibody was detected at birth and where the sow had been infected prior to foetal immunocompetence (D35 (Ab −ve) 7.8 log_10_ copies/mL; D55 (Ab −ve) 8.4 log_10_ copies/mL) (Figure 2). For those animals seropositive at birth, the infection gradually cleared over time, although low-level viraemias were still detected at 75 days of age in some animals. At around 28 days of age when maternal antibodies started to wane (Figure 3) the viral load in serum started to rise for the pigs born seronegative in the D35 and D55 groups (Figure 2). By day 120 viral RNA could no longer be detected in the serum of the four remaining D55 (Ab +ve) pigs (data not shown).
- Oropharyngeal swabs: the mean quantity of viral RNA detected on oropharyngeal swabs at birth ranged from 6.5 (D90) to 7.2 log_10_ copies/swab (D55 Ab −ve & Ab +ve) (Figure 4). Through to 75 days of age the amount of RNA detected on oropharyngeal swabs remained elevated for the D35 (Ab −ve) and D55 (Ab −ve) groups (Figure 4). In contrast, for all other groups, the amount of RNA decreased over time, reducing most rapidly for the D75 and D90 animals. By day 120 viral RNA could no longer be detected on oropharyngeal swabs of the three D55 (Ab +ve) pigs sampled (data not shown).
- Rectal swabs: the mean level of viral RNA detected on rectal swabs at birth ranged from 6.3 (D90) to 7.1 log_10_ copies/swab (D55 Ab −ve) (Figure 5). As observed for oropharyngeal swabs, the amount of RNA detected on rectal swabs remained elevated for the D35 (Ab −ve) and D55 (Ab −ve) animals through to day 75. In contrast, for all other groups where the pigs were seropositive at birth, the amount of RNA decreased over time, reducing most rapidly for the D75 and D90 animals. By day 120 viral RNA could not be detected on rectal swabs from the four remaining D55 (Ab +ve) pigs (data not shown). Three animals from the D55 (Ab −ve) group were followed for >6 months (8-01, 8-05 and 10-01). Pig 10-01 appeared to seroconvert between 42 to 49 days old, with a marked reduction in viral load in serum and on rectal swabs from around 84 days of age, and oropharyngeal swabs on day 98 (Figure 6A). Virus was not detected in the serum of 10-01 at time of euthanasia on day 189, but low levels of viral RNA were still detected on oropharyngeal and rectal swabs. Pigs 8-01 and 8-05 seroconverted between 127 and 190 days of age (Figure 6B,C) after which the viraemia was cleared. The amount of virus detected on oropharyngeal and rectal swabs decreased more rapidly after seroconversion for 8-01 compared with 8-05 (Figure 6B,C).
- Mummified foetuses: the mean quantity of viral RNA detected on swabs of the internal organs of mummified foetuses was 5.6 log_10_ copies/swab for D35 (n = 13; range 4.9–6.9 log_10_ copies/swab), 5.6 log_10_ copies/swab for D75 (n = 2; range 5.5–5.7 log_10_ copies/swab) and 3.0 log_10_ copies/swab for D90 (n = 1).
- Tissues: at birth and in the first 10 days of life, virus was readily detected in all tissues collected from infected pigs in groups D35, D55 and D75 (Table 1). For group D90, virus was not always detected in tissues sampled at birth but was most likely to be detected in tonsillar swabs and from lymph nodes and heart. For all groups, in the first 10 days of life, the greatest quantity of viral RNA was detected from the tonsils (range of means 5.8 to 7.2 log_10_ copies/swab) and the brain (range of means 4.8 to 7.0 log_10_ copies/swab). Subsequently, the amount of Bungowannah viral RNA detected in tissues decreased over time and the proportion of tissues in which viral RNA was detected decreased in those groups where the pigs were seropositive at birth. This decrease occurred most rapidly for the D75 and D90 animals (Table 1). There were only two D35 (Ab +ve) animals identified and by the end of the study (day 75) Bungowannah viral RNA was either no longer detected in tissues or was at low levels, with the highest levels in lymph nodes followed by tonsil (Table 1). In contrast, for D35 (Ab −ve) virus was detected at all time-points for all animals and remained elevated in all tissues with highest quantities detected from tonsil, and in lymph nodes and brain. For the D55 (Ab −ve) group the amount of RNA detected in tissues was generally higher for the first two time-points compared with D55 (Ab +ve) although the number of animals sampled was low. An extensive range of tissues was collected from the presumptively PI pigs (8-01 and 8-05) that were followed until 11 months of age (Table 2). The quantity of Bungowannah virus RNA detected in epididymal semen (9.8 log_10_ copies/mL) is the highest amount of viral RNA detected in any sample collected throughout the course of the study.
- Urine: urine was collected directly from the bladder of six D35 (Ab −ve) animals at necropsy at between 57–77 days of age in with a mean 6.2 log_10_ copies/mL (range 5.1–6.9) of viral RNA detected. Virus was also detected in the urine of four D55 (Ab +ve) pigs between 80–90 days of age (mean 4.4 log_10_ copies/mL) but by day 117 viral RNA was no longer detectable in three. In contrast, Bungowannah virus RNA was only detected in the urine of 2/12 animals from D90 in low quantities when euthanased between 19 and 22 days of age (2.7 log_10_ copies/mL).

#### 3.1.3. Piglets—Serology

The antibody titres of surviving pigs in each of the challenge groups between age 0 to 75 days are presented in Figure 3. Pigs that were known to have fed and were seropositive were excluded from the day 0 antibody titre calculations. The results can be broadly divided into four groups:

- Animals are detected with antibody prior to suckling (or are presumably in the process of seroconverting – D90). The percentage of seropositive (titres ≥10) pigs for each challenge group seropositive was 4% (D35), 82% (D55), 98% (D75) and 50% (D90). The highest antibody titres were detected in those litters infected earliest in gestation: D35 (mean = 1280; n = 2), D55 (mean = 654; n = 32), D75 (mean = 164; n = 45) and D90 (mean = 27; n = 50). After ingestion of maternal antibodies, the antibody titre rises and stays elevated for the remainder of the study (D35 (Ab +ve); D55 (Ab +ve); D75; D90).
- Animals are seronegative at birth; antibody levels increase with ingestion of maternal antibody and then gradually wane with the animal becoming seronegative again at around 40–60 days of age (D35 (Ab −ve)).
- Animals are seronegative at birth (D55 (Ab −ve); n = 7). These animals have the same serological profile as the D35 group after the ingestion of colostrum. At a variable time after losing maternal antibody these animals seroconvert (10-01 at between 42–49 days of age; 8-01 and 8-05 between 127 and 190 days of age; Figure 6). Note that the seroconversion of 10-01 has resulted in the large box plot for D55 (Ab −ve) from day 42 onwards, as the other two animals in the group (8-01 and 8-05) were seronegative at these time-points.
- Uninfected (seronegative) pigs born to an infected dam; antibody levels increase with ingestion of maternal antibody and then gradually wane with the animal becoming seronegative again at around 60–70 days of age.

### 3.2. Transmission Study

**Figure 7:**
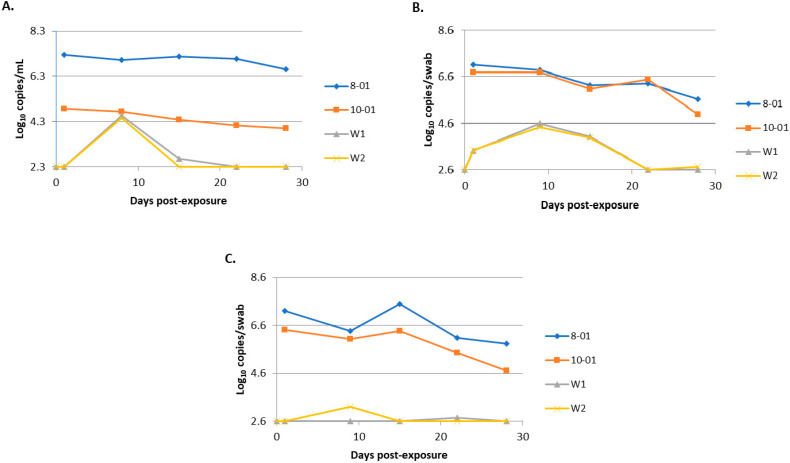
Comparison of the quantity of virus detected in serum (**A**), oropharyngeal swabs (**B**) and rectal swabs (**C**) for persistently infected (PI) animals (8-01 and 10-01) and transiently infected in-contact animals (W1 and W2).

Bungowannah viral RNA was detected on oropharyngeal swabs collected from the introduced pigs at 24 h after their entry to the room/pen containing the presumptively PI animals. Peak viraemia and viral shedding were detected at 8 (serum) or 9 (oropharyngeal and rectal swabs) days post-introduction. Both introduced pigs seroconverted between days 8 and 15 with a titre of 5120 recorded at day 15. Figure 7 illustrates the differences in levels of viraemia and shedding observed between the PI and transiently infected pigs in this study. The differences in viral load detected on oropharyngeal swabs were >2 log_10_ copies per swab higher for the PI pigs compared to the transiently infected in-contact pigs at peak shedding on day 9. This difference was more than 10-fold higher for the rectal swabs. At the time of peak viraemia in the transiently infected pigs the viral load was equal to that of pig 10-01 but still >2 log_10_ copies/mL less than that detected in 8-01.

## 4. Discussion

This study demonstrates that the porcine foetus can become infected with Bungowannah virus following intranasal challenge of the dam and characterises the virological and serological responses following in utero infection of the porcine foetus at different stages of gestation. Persistent infections, as described for other pestiviruses, were observed, as was a chronic infection state where animals that had been presumed to be PI animals later seroconverted and cleared the viral infection. Transmission of Bungowannah virus infection from chronically infected pigs to naïve pigs was readily achieved, with evidence of virus transfer within 24 h.

Preliminary investigations to identify the causative agent of the porcine myocarditis syndrome utilised a direct foetal inoculation route [25]. In the current study, the goal was to determine the outcomes using a natural route of infection at different stages of gestation. Transplacental infection was successfully achieved following intra-nasal exposure of the dam in 87% (20/23) of the litters. In addition, this outcome was not affected by the stage of gestation of the sow at the time of challenge.

Regardless of the stage of gestation that infection occurred, Bungowannah virus was detected in the serum, body fluid and excretions of infected pigs at birth and this was unrelated to the presence of precolostral Bungowannah virus-specific antibody in these animals. Generally, there was minimal difference in virus shedding between the pigs in the different challenge groups at birth, although the mean and median were lowest for the D90 group. The highest viral loads in serum or body fluid were recorded in the D35 (Ab −ve) and D55 (Ab −ve) groups. Over the course of the study it was noted that viral shedding reduced more quickly the later in gestation that the dam was challenged for those pigs which were seropositive at birth or in the D90 group. For those pigs seronegative at birth (D35 and D55 groups), viral shedding remained elevated throughout the course of the study. The exception was those animals that seroconverted after 6–7 weeks (10-01) and between 18 and 28 weeks (8-01 and 8-05) after which viral shedding gradually decreased, although the reduction varied between pigs. In contrast, the viral load in serum declined for all challenge groups after the ingestion of colostrum, but, as maternal antibodies waned in those animals that were seronegative at birth (D35 and D55), the viral load in serum increased as has been described for CSFV [13,14,15]. Despite animals 8-01, 8-05 and 10-01 seroconverting, it still took approximately 3 months for the viraemia to clear after this event.

It is interesting to compare our results with experimental studies of classical swine fever virus in the porcine foetus which were conducted 40–50 years ago with the less sensitive technologies of virus isolation and antigen detection by immunofluorescence [13,14,21,29]. While test sensitivity may affect the direct comparison of the proportion of animals infected with CSFV at birth compared to Bungowannah virus, the findings are similar. Virus could be detected in a proportion of all pigs born following infection with CSFV both pre- and post-immunocompetence as we have observed. Van Oirschot [13] also described congenitally infected pigs that cleared their CSFV infection by 2 weeks of age. The proportion of pigs that recovered increased the later in gestation that infection took place and was also associated with a low viral load in serum at birth. While precolostral CSFV neutralising antibodies were only detected in one animal (sow infected at 90 days of gestation), these virology findings are similar to those observed for congenital Bungowannah virus infections.

The pig foetus is able to mount a humoral immune response to Bungowannah virus following in utero infection. The highest antibody titres at birth were observed in those pigs that seroconverted and were the progeny of sows challenged at D35 and D55. Only one pig at D70 had no detectable antibody and it was stillborn. Antibody titres in the D90 group were generally low at birth presumably due to the short interval between infection and birth. However, the detection of viraemia indicates that all pigs in the D90 litters were infected prior to birth. Pigs infected post-natally generally seroconvert from 12–14 days post-infection [23]. Therefore, based on the D90 data, it is considered that transplacental and subsequent foetal infection had occurred within 8–10 days of intra-nasal exposure of the sow.

Unfortunately, farrowings were not monitored throughout the night and in some cases pigs were able to feed prior to precolostral blood samples being collected. While not optimal, we were still able to identify pigs in the D35 and D55 groups that did and did not mount a humoral immune response in utero and demonstrate that the time of gestation at which infection occurs has an influence on the ability of the porcine foetus to mount a humoral immune response to Bungowannah virus. The porcine foetus becomes competent to respond to infection with CSFV between days 70–90 of gestation and the serological findings of this study would suggest that the timing is similar, although likely closer to 70 days for Bungowannah virus [13,14,21]. The failure of some pigs in the D35 and D55 litters to seroconvert is indicative of immunotolerance, which occurs following infection prior to foetal immunocompetence and is recognised with other in utero pestivirus infections [5,13,14,21]. In the D55 group it was in Litters 8 and 10, that were challenged at 56 and 55 days of gestation, respectively, that the birth of litters with a mix of both seronegative and seropositive pigs resulted. A previous study [25], suggested that direct in utero transmission of Bungowannah virus to adjacent foetuses is probable and this has also been suggested for CSFV [22]. We speculate that the mixed outcomes of seronegative and seropositive pigs at birth in the D55 and to a lesser extent the D35 group are a consequence of the timing of foetal infection following transplacental transmission, and timing of any subsequent infections that result from direct in utero transmission.

While the current study did not measure neutralising antibody, the findings have similarities to those of Frey et al. [21] who did not detect neutralising antibody against CSFV in progeny at term when pregnant gilts were infected with CFSV at 65–67 days of gestation. In contrast, 3/4 (75%) of foetuses from gilts infected at 85 days of gestation had neutralising antibodies at term; additionally, antibodies were only detected in 2/6 (33%) of infected pigs when infection occurred after 94 days of gestation. As these foetuses were collected via hysterectomy at term it is not possible to determine if the failure to detect a neutralising antibody response is due to insufficient time between infection and sampling or the result of immunotolerance. Interestingly, Van Oirschot [13] with one exception did not detect precolostral antibodies in any pigs following challenge of the dam with CSFV at 40, 65 or 90 days gestation although the ability of some pigs (highest percentage at 90 days) to clear their infection by 2 weeks of age indicates they were not immunotolerant. Further comparison of antibody titres as measured by PLA and the virus neutralisation test may clarify the nature of the humoral immune response that develops in the period immediately following the development of immunocompetence compared with later in gestation.

The results also suggest that the cell-mediated immune response to Bungowannah virus may be suppressed if the foetus is infected before 90 days gestation or that the antibody response at this time is non-neutralising. In those foetuses from dams infected at 75 days of gestation or earlier that mounted an antibody response, even after ingestion of colostrum, the time taken for these animals to clear the infection was generally delayed compared to those from dams infected at 90 days gestation.

The identification of viraemic pigs that were seronegative following infection of the sow with Bungowannah virus at 35 or 55 days of gestation suggests that persistent infections may result. Persistent pestivirus infections occur following in utero infection of the foetus prior to it becoming immunocompetent and have been described following infection prior to day 70 to 90 of gestation for CSFV, bovine viral diarrhoea virus (BVDV) and Border disease-like viruses in the pig [13,14,30,31]. Despite two pigs in D35 not becoming PI, infection at this stage of gestation results in a high probability of occurrence. By day 55 of gestation the likelihood of maternal infection resulting in persistent infections is declining and may result in a mixed outcome for foetuses within that litter. Based on the results of this study and direct foetal inoculation [25], infection of the pregnant pig after approximately day 60 of gestation (approximately day 70 for foetal infection) seems unlikely to result in persistent infections.

At birth, PI animals could only be differentiated in the laboratory by their seronegative status. While the mean viral load in serum at birth was higher than for those animals born seropositive, individual animal variation was sufficient to prevent the titre of viraemia being a distinguishing feature. Furthermore, it was not until maternal antibodies began to wane from approximately 4–5 weeks of age in the PI pigs that the viral load in serum was higher than the non-PI animals. In contrast, the viral load on oropharyngeal and rectal swabs from PI animals remained elevated throughout the study period but the viral load on swabs for those animals seropositive at birth declined from 3 weeks of age. PI pigs from the D35 group grew poorly and died or were euthanased by 75 days of age [24]. While those from the D55 group were stunted [24], they had survived longer.

Three pigs, from the D55 group (8-01, 8-05 and 10-01) were monitored over a 6 to 11 month period to assess their virological and serological status over time and for any abnormal clinical signs [24]. Unexpectedly, these animals that appeared to be PI seroconverted at varying times during this observation period. This phenomenon has been described rarely in pigs infected with BVDV or border disease virus (BDV) [30,31]. Like these previous studies with BVDV and BDV, the Bungowannah virus viraemia ceased after seroconversion but virus could still be detected in many tissues for a further 5–6 months and at high levels for the female (8-05). For the male (8-01), the highest viral load was 9.8 log_10_ copies/mL in epididymal semen and was generally above 6.0 log_10_ copies/mL for other testicular samples (Table 2). Such high virus levels have also been observed in a PI bull [32] and in the pig 8-01 presumably remained high following seroconversion due to the immunologically privileged status of the testes. In contrast, for the female (8-05) the highest viral loads were detected in lymphoid tissues (tonsil and lymph node) (Table 2). It is also of interest to note the high quantity of Bungowannah virus detected in the seminal fluid of the male in the absence of spermatozoa [24]. This has also been described in a boar PI with BVDV [32]. The mechanism for the failure of virus to be cleared from tissues at the same time as the resolution of the viraemia is not clear but may be related to clearance from infected cells being dependent on cell-mediated immunity rather than just the presence of antibody and its inability to access immunologically privileged sites.

Chronic infections resulting from in utero infections with pestivirus have not been reported in ruminants with one exception of BDV in a sheep [17]. It seems probable that the mechanism for development of the chronic infections observed in pigs are related to differences in the time required for maturation of the immune system of ruminants and pigs given that the porcine foetus becomes immunocompetent at a proportionately later stage of gestation compared to ruminants.

While the two chronically infected pigs followed to 11 months of age were quite stunted and would not have been selected as breeding animals [24], they have the potential to be more important in the ongoing transmission of Bungowannah virus than the PI pigs that die early in life. To this end, we were able to show that Bungowannah virus is readily transmitted by these animals to naïve pigs in close proximity.

At least 100–1000 times more Bungowannah virus RNA was detected on oropharyngeal and faecal swabs from pigs at birth in this study compared with peak viral shedding of transiently infected animals [23]. The difference in quantity of virus shed by persistently/chronically infected animals in oropharyngeal swabs compared with peak shedding by transiently infected animals remained approximately 100 times higher until seroconversion was detected in the chronically infected animals. In addition, while the virus load in faeces is negligible in the transiently infected pig, faeces along with urine appear to be an important route of shedding in the PI pig (100–1000 times greater quantity), emphasising the role of PI animals in the epidemiology of pestivirus infections. Placental and vaginal secretions also appear to be an important source of virus for environmental contamination and ongoing transmission and has previously been recognised for BVDV [33]. High quantities of viral RNA could be detected in the placental and vaginal secretions of recently farrowed animals that carried infected foetuses. Although the quantity detected decreased rapidly over the first 5–7 days after farrowing, these reproductive materials could provide a significant source of virus in a population of pregnant animals to sustain transmission cycles even in the absence of a PI animal. This study indicates that all recently farrowed Bungowannah virus infected litters are also potentially adding large quantities of virus into the environment. Further studies are required to determine if these animals are shedding an infectious virus and, therefore, a source of virus for ongoing transmission, although given the quantities of Bungowannah virus RNA detected on oropharyngeal swabs we believe this is highly likely to be the case.

Finally, examination of the virus loads in tissue samples indicates that Bungowannah virus is widely disseminated in the porcine foetus at birth and provides an insight into sample selection for diagnostic purposes from live and dead animals. While the virus could usually be detected in a wide range of samples collected at birth, the optimal tissues to sample for virus detection were serum/body fluid, oropharyngeal swabs, tonsils and lungs. In older animals, virus can also be readily detected in a wide range of samples from PI animals, although serum, oropharyngeal swabs and lymph nodes are preferred for the greatest chance of detection of infection in animals greater than 10 days of age that may not be PI. The high quantity of virus detected in tonsillar samples suggests that the tonsils are the primary source of Bungowannah virus found in oropharyngeal secretions.

## 5. Conclusions

The results of this study provide a unique insight into the biology of Bungowannah virus infections in the porcine foetus and the subsequent pre- and post-weaning period following infection at different stages of gestation. While the birth of PI animals was expected based on our knowledge of pestivirus infections in other species, the birth of chronically infected pigs that later went on to seroconvert was unexpected. This study provides further information about how the porcine foetus responds to in utero pestivirus infections.

## Figures and Tables

**Table 1:** Quantity of Bungowannah virus RNA detected in selected tissues at four time periods after birth in challenge groups D35, D55, D75 and D90.

		D35 (Ab +ve)			D35 (Ab −ve)			D55 (Ab +ve)			D55 (Ab −ve)			D75			D90		
Tissue	Age	No. +ve (%) ^a^	Mean ^b^	Range ^b^	No. +ve (%)	Mean	Range	No. +ve (%)	Mean	Range	No. +ve (%)	Mean	Range	No. +ve (%)	Mean	Range	No. +ve (%)	Mean	Range
Tonsil	0–10				28 (100%)	7.1	6.2–8.3	10 (100%)	7.2	6.3–7.8	2 (100%)	7.2	7.0–7.5	30 (100%)	6.7	5.0–7.8	24 (96%)	5.8	ND–7.7
	11–30				4 (100%)	6.7	5.2–7.6	5 (71%)	4.4	ND–7.1	2 (100%)	6.5	6.4–6.6	3 (75%)	4.0	ND–6.4	1 (5%)	2.6	ND–2.8
	31–60				5 (100%)	6.9	6.4–7.4							8 (44%)	2.9	ND–4.3	1 (8%)	2.6	ND–2.8
	>60	1 (50%)	3.3	ND–4.0	3 (100%)	7.0	6.5–7.4	10 (48%)	3.2	ND–6.8	0 (0%)	<2.6	NA						
Lymph	0–10				31 (100%)	5.3	4.4–6.3	10 (100%)	5.4	4.5–6.4	2 (100%)	4.5	3.9–5.2	34 (100%)	5.3	3.9–6.5	26 (100%)	4.7	3.2–6.4
Node	11–30				4 (100%)	4.5	3.4–5.7	7 (100%)	5.1	2.8–6.0	2 (100%)	5.5	5.0–6.1	4 (100%)	4.8	3.7–5.4	12 (55%)	3.6	ND–5.6
	31–60				5 (100%)	5.3	4.5–6.6							12 (71%)	3.9	ND–6.3	10 (77%)	3.9	ND–5.4
	>60	2 (100%)	4.2	4.0–4.5	3 (100%)	6.0	5.7–6.2	17 (77%)	4.5	ND–6.5	1 (100%)	6.5	NA						
Spleen	0–10				30 (100%)	5.9	4.5–7.3	10 (100%)	5.2	4.7–5.8	2 (100%)	4.9	4.7–5.0	34 (100%)	5.4	3.6–6.9	18 (69%)	3.6	ND–5.2
	11–30				4 (100%)	4.5	3.4–5.9	7 (100%)	3.8	2.9–4.7	2 (100%)	4.3	3.8–4.8	4 (100%)	3.5	2.8–4.6	1 (5%)	2.6	ND–2.8
	31–60				5 (100%)	4.6	3.8–5.8							9 (50%)	3.1	ND–4.4	0 (0%)	<2.6	NA
	>60	1 (50%)	2.8	ND–3.1	3 (100%)	5.8	5.3–6.4	14 (67%)	3.3	ND–4.9	1 (100%)	3.9	NA						
Thymus	0–10				29 (100%)	5.6	4.1–6.4	10 (100%)	5.2	4.3–5.8	2 (100%)	4.9	4.8–5.1	32 (100%)	5.4	3.3–6.8	19 (79%)	4.1	ND–5.7
	11–30				4 (100%)	4.3	3.0–5.1	7 (100%)	3.8	2.9–4.9	2 (100%)	4.5	4.5–4.6	1 (25%)	2.9	ND–3.7	2 (10%)	2.6	ND–3.2
	31–60				5 (100%)	4.6	4.0–5.4							7 (39%)	2.8	ND–3.9	1 (8%)	2.6	ND–3.0
	>60	0 (0%)	<2.6	ND	3 (100%)	5.2	4.7–5.5	9 (41%)	3.0	ND–4.5	1 (100%)	3.0	NA						
Lung	0–10				31 (100%)	6.4	5.1–7.6	10 (100%)	6.0	5.2–6.8	2 (100%)	6.2	6.2–6.2	33 (100%)	6.5	4.4–8.2	21 (81%)	4.2	ND–6.2
	11–30				4 (100%)	4.8	4.2–5.6	7 (100%)	3.8	2.7–5.5	2 (100%)	4.7	4.5–4.9	4 (100%)	3.9	3.6–4.2	1 (5%)	2.6	ND–2.9
	31–60				5 (100%)	4.8	4.3–5.5							11 (61%)	3.4	ND–6.8	2 (15%)	2.7	ND–3.1
	>60	0 (0%)	<2.6	ND	3 (100%)	5.0	4.4–6.1	12 (57%)	3.2	ND–5.5	1 (100%)	4.1	NA						
Heart	0–10				31 (100%)	6.1	4.3–7.2	10 (100%)	6.0	4.9–7.0	2 (100%)	5.6	5.5–5.8	34 (100%)	6.0	4.2–7.9	26 (100%)	5.4	3.9–6.9
	11–30				4 (100%)	4.3	3.7–5.2	7 (100%)	3.7	2.6–4.8	2 (100%)	4.2	4.2–4.2	4 (100%)	3.4	2.9–4.2	8 (36%)	2.9	ND–4.1
	31–60				5 (100%)	4.2	3.4–5.2							4 (22%)	2.8	ND–4.3	4 (31%)	2.7	ND–3.1
	>60	0 (0%)	<2.6	ND	3 (100%)	4.3	3.8–5.1	7 (32%)	2.9	ND–4.9	0 (0%)	<2.6	NA						
Intestine	0–10				31 (100%)	5.4	3.4–7.4	10 (100%)	4.3	3.3–5.7	2 (100%)	4.6	4.4–4.8	34 (100%)	5.0	2.6–6.8	17 (68%)	3.7	ND–5.5
	11–30				4 (80%)	4.4	ND–5.4	3 (43%)	3.0	ND–4.5	1 (50%)	3.8	2.6–5.1	3 (75%)	3.5	ND–4.3	2 (10%)	2.7	ND–3.8
	31–60				5 (100%)	6.2	4.2–7.1							3 (17%)	2.7	ND–3.7	4 (31%)	2.8	ND–3.3
	>60	0 (0%)	<2.6	ND	3 (100%)	5.7	5.2–6.1	10 (45%)	3.1	ND–6.2	1 (100%)	4.5	NA						
Brain	0–10				32 (100%)	7.0	6.1–7.8	10 (100%)	6.5	5.4–7.7	2 (100%)	6.8	6.5–7.2	30 (100%)	6.3	5.2–7.4	23 (92%)	4.8	ND–6.2
	11–30				4 (100%)	6.1	5.8–6.5	7 (100%)	5.3	4.4–6.1	2 (100%)	5.9	5.8–6.0	4 (100%)	4.3	3.8–5.0	4 (18%)	2.9	ND–4.8
	31–60				5 (100%)	6.0	5.5–6.5							14 (78%)	3.5	ND–4.5	8 (62%)	3.1	ND–3.9
	>60	1 (50%)	3.1	ND–3.7	3 (100%)	6.4	5.8–7.2	17 (77%)	4.0	ND–5.9	1 (100%)	3.3	NA						

^a^ Percentage positive of total animals sampled at that time–point; ^b^ Log_10_ copies/swab; ND = Not detected.

**Table 2:** Quantity of Bungowannah virus RNA detected in selected tissues and fluids from pigs 8-01 and 8-05 (D55 Ab −ve) at 11 months of age.

Animal ID	Sample	Viral Load ^a^	Animal ID	Sample	Viral Load ^a^
08-01	Tonsil	4.74	08-05	Tonsil	7.46
	Lymph node	4.82		Lymph node	7.31
	Spleen	4.36		Spleen	4.93
	Thymus	ND		Thymus	ND
	Brain	ND		Brain	4.18
	Heart	4.25		Heart	3.78
	Lung	ND		Lung	ND
	Intestine	4.78		Intestine	6.34
	Urine	6.9		Urine	5.8
	Kidney	ND		Kidney	3.7
	Epididymal semen	9.8		Ovarian follicular fluid	ND
	Seminal fluid	3.4		Cervix	ND
	Bulbourethral gland	5.8		Ovary	2.8
	Epididymis (head)	6.2		Uterus	4.3
	Epididymis (tail)	8.0		Vagina	5.2
	Prostate	4.1			
	Seminal vesicle	ND			
	Testis	6.3			

^a^ Log_10_ copies/mL or swab; ND = Not detected.

## Supplementary Materials

Supplementary materials can be found at https://www.mdpi.com/1999-4915/12/6/691/s1. Summary of the number of animals from which serum or body fluid was collected at each time-point for Bungowannah virus PCR (Table S1).
