# Supplementary material for: The Outcome of Porcine Foetal Infection with Bungowannah Virus Is Dependent on the Stage of Gestation at Which Infection Occurs. Part 1: Serology and Virology
Source: Viruses. 2020 Jun 26;12(6):691. doi: 10.3390/v12060691 (PMC7354495; doi:10.3390/v12060691)
Supplement: Supplementary file 1 [file viruses-12-00691-s001.pdf]

Table S1. Summary of the number of animals from which serum or body fluid was collected at each time-point for Bungowannah virus PCR.

	**Day 0**	**Day 7**	**Day 14**	**Day 21**	**Day 28**	**Day 35**	**Day 42**	**Day 49**	**Day 56**	**Day 65**	**Day 75**	**Day 85**	**Day 95**	**Day 120**	**Day 190**	**Day 239**	**Day 329**
	(0–1)	(2–9)	(10–17)	(18–24)	(25–31)	(32–38)	(39–45)	(46–52)	(53–60)	(61–70)	(71–80)	(81–90)	(91–100)	(101–130)	(189–190)	(239)	(329)
**D35 (Ab +ve)**	2	2	2	2	2	2	2	2	2	2	2						
**D35 (Ab −ve)**	48	17	12	9	9	8	6	6	6	2	1						
**D55 (Ab +ve)**	38	28	28	26	18	18	18	18	18	12	7	4	3	4			
**D55 (Ab −ve)**	7	5	5	5	3	3	3	3	3	2	3	3	3	3	3	2	2
**D75**	55	25	22	15	22	18	15	16	12								
**D90**	57	47	35	33	20	13											
